# Artificial intelligence in assisted reproduction: psycho-emotional repercussions

**DOI:** 10.5935/1518-0557.20240065

**Published:** 2024

**Authors:** Rose M. Massaro Melamed

**Affiliations:** 1 Psychologist - Fertility Medical Group - São Paulo, Brazil

The advancement of technologies and the influence of these advances on man’s relationships with different aspects of life are undeniable, these changes can be perceived in all environments (Machado *et al*., 2011). In the health sector, we hear about artificial intelligence, a new category of scientific studies focused on equipment that optimizes care, diagnoses, and treatments in health centers.

As an example of this improvement process, in assisted reproduction, this resource is used in the search for answers for treatments and clinical diagnoses, in embryology laboratories, which may focus on the diagnostic analysis of aspects relating to the patient’s fertility, and the development of the embryo before being transferred to the mother’s womb.

However, when we think about man’s interaction with this progress and its practical results in this process, it is up to us to think about the issue that emerges, about the clarity of how information will be conveyed and interpreted; for all intents and purposes, a new relationship perspective is created, and a sociocultural space is established to promote development in society.

For as long as we have known, technology has been helping our daily lives by enabling us to obtain accurate and faster results, such as communication between people in real-time and faster diagnoses, which facilitates the healing process, among many other things.

Focusing our attention on the assisted reproduction process, the development of techniques was expanded, concerning the possibilities and development of parenthood, circumstances never thought of before, regardless of the social, scientific, and technological advances in biotechnology and medicine that have occurred in recent decades, Generally, the inability to conceive, carry and give birth to the desired child can be experienced as a stressful and lonely situation, as several authors have already stated (Cousineau & Domar, 2007; Melamed, 2018).

It is known that infertility and its diagnosis not only cause physical suffering, but also a psycho-emotional one, since having children is part of most people’s imagination and seems to be the most “natural”. Even though we are living in a time where the limits imposed on certain issues have been changed and, the economic independence of women created favorable conditions for them to separate from their husbands/partners, nowadays sexual life is disconnected from motherhood, occupying different spaces, the right to choose the most appropriate time for pregnancy was thus provided.

For Chatel (2008), the social change in the place of women was called “the shock of our century”. It made it possible to medically control births and separate the sexual act from procreation, but the desire for a child remains alive in most people, despite there being more cases of infertility diagnosed.

Upon receiving a diagnosis of infertility, the person feels prevented from achieving the goal of becoming pregnant at the desired time; and, in this circumstance, resorting to assisted reproduction processes is the resource found for the movement in search of pregnancy and a child at home.

*But what child are we talking about, if biologically it does not yet exist?*

*What we have at this moment is just the desire for the long-awaited child.*

In terms of subjective experience, the bond with this heir heads the relationship, and so we reflect that it is not necessary to be inside a woman’s womb to be considered her child, and it doesn’t matter whether we talk about an embryo, a newborn, or a baby. What matters is the relationship that is established in an intense, personal way, that “is in the psyche”, that is, that is in the affection of the parents (Melamed & Santos, 2021).

The desired child, already conceived in the imagination and thus imagined in its most complete form of being, is sometimes expected, in a prolonged pregnancy, which begins not at the end of the last menstrual cycle, but at the first injection when treatment begins. And, thus, in terms of subjective experience, the connection with the possible offspring has already come into existence, although several stages constitute the course of this process and possible emotions can emerge, depending on the activation of the psychic contents then mobilized.

When encountering mobilized emotional psychic contents, some patients demonstrate the need to obtain information about the development of each phase they are in during their treatments. The stimuli for the attitudes described demonstrate a certain fear or degree of insecurity in separating from the object of love.

When the person or patient demonstrates the need to take control of everything, this behavior may be related to the fear of internal ‘lack of control’, that is, the essential characteristic of attachment tends to keep the subject close to the object and, faced with any possibility of separation, causes changes. These are the situations in which an intruder tries to take the puppies away from a mother, arousing negative reactions, called emotional triggers (Bowlby, 2006).

To understand this process in psychic terms, we turn to Winnicott (Silva, 2016), who highlights the importance of investigating when babies were conceived psychically so that they can later investigate when they were conceived biologically or physically. In other words, before a baby exists, it must have been desired or, at least, created in the inner fantasy of one of the parents. The biological child normally comes into existence after the approach of a man and a woman, who at a certain point in the life cycle, through sexual intercourse, give rise to the offspring; With the development of biotechnology, this order could be changed by dispensing with coitus, allowing conception in the laboratory.

We clarify that the situation or experience that has the power to change the mood and trigger physical symptoms and interfere with behavior, emotions, and the functioning of the organism makes life more complicated and requires adaptation, therefore, when a person has a growing need to take control of things around them, the fact that the body does not function as planned makes them feel extremely helpless; or it can remain veiled and protected and at the same time, enjoy between inside and outside and be found, in a creative way (Mota, 2013).

Considering the aspects involved in this context, we understand that the possibility of giving new meaning to experiences and psychological suffering can arise from knowledge about the disease, its possible causes, methods of control, and the side effects of medications that can help with adherence to treatment in a variety of contexts (Mota, 2013). From the initial medical appointment to the result of a procedure, there are several moments when it will be necessary to clarify, inform, welcome, discuss, and reflect on the various issues involved in this process.

The beginning with the presence, or even the possibility of a diagnosis, leads the individual to confront the risk of suffering, pain, and death of a dream (Quayle, 2019). Among the repercussions of this rupture is the possibility of compromising psychological well-being; which causes a breakdown, a hiatus, and a narcissistic wound; expressed in the question of many patients “Why is this happening to me?” “Why me?”.

Among patients’ narratives about the difficulty of having a favorable prognosis, we often observe that this is burdened by the status of failure as if the process could have been avoided by them (Moretto, 2019). Faced with this circumstance of being responsible for their condition, these people present themselves to the psychologist in distress.

Among the possibilities of action of the mental health professional, faced with what we call suffering, resulting from what is felt as failure, we have Psychoeducation as a resource used to minimize symptoms, since the objective of this technique is to support the person in their needs, exposing them to knowledge about their disease and/or diagnosis and the entire treatment process. Using the resources of psychology, pedagogy, and person-centered care, the professional needs to connect with the patient in understandable language and provide the necessary information (Straube *et al*., 2018).

When referring to the transmission of information to the patient, an aspect to be taken into consideration concerns the development of biotechnology and the use of artificial intelligence in attention to specific aspects. The particularities of each case, notably the possibility of contributing to humanization and better integration in communication with the patient, are points to reflect on since there are stages in which the patient is separated from what is happening, for example in embryonic development, in which the handling of genetic material is carried out by the embryologist, the responsible professional within a laboratory.

Many patients do not know exactly what the embryologist does in their daily lives, a fact that generates curiosity, and in more extreme cases of insecurity, the “ideal would be to take the would-be parents into the laboratory” where the genetic material is handled. This professional also monitors its development during the pre-implantation period, that is, before it is transferred to the uterus.

The work of embryologists “has become easier”, through the refinement of techniques and the use of increasingly modern equipment for embryo evaluation. The time-lapse system (TLS), incorporated into modern incubators, allows continuous monitoring of embryonic development, as it is a dynamic and interactive approach, with greater precision for the selection and definition of embryo transfer. Simas *et al*. (2020) The acquired images are processed and organized into a short film, which uses the computer screen connected to the software to evaluate embryonic development, in addition to making it possible to make images and videos of embryos available to patients in real-time. It is a way of engaging future parents in this stage of treatment, reducing the possible discomfort at this time.

There are indisputable facts, such as what concerns a biological entity, which carries within itself a new project “a potential child”, which each person experiences in a real and imaginary way. But, even though they can be observed by parents, professionals still need to welcome those involved to help them manage and understand the biopsychosocial dimensions of the moment they are going through.

Regarding the patient’s perception, if we think about these cells, we observe that they are the ones that will give rise to the child, already existing in the imagination and with whom the emotional bond is established, therefore, adversity in the evolution of the embryo will awaken feelings of regret, which can trigger intense feelings, especially when the unfavorable diagnosis is “totally unknown”, in addition to being able to re-edit the initial difficulty.

*And once again we ask ourselves: which child are we talking about?*

Based on the theory of object relations, made famous by the English School of Psychoanalysis, some authors began to rethink the baby from preand post-natal life and how these experiences influence their psyche.

*Parents began to consider the baby as a person, seeing in him much more than what was there - a little man or a littlewoman. This was initially rejected by science, which stated that the child is not a small adult, and for a long time observers objectively considered children as very little human beings until they began to speak. Recently, however, it has been discovered that babies are, in fact, human, albeit suitably infantile (Silva, 2016).*

We could infer that, for our patients, the fact following embryonic development would minimize the initial tension caused by waiting for the embryo’s evolutionary process, this being, therefore, an intermediate area of the experience that can alleviate the tension related to internal and external reality.

This intermediate area would therefore be a space between the “imagined child” and the “child ready to be transferred to the uterus”; on the other hand, when we reflect on possible unfavorable results concerning embryonic development, a fact that would affect this negative experience, similar to cases of early loss of a child who was not born, it is a somewhat marginal experience, which occurs on the periphery of parenthood and its rites (Quayle, 2019).

The experience of loss, generally without a place for expression and listening, can be devastating, increasing fears, anguish, loneliness, isolation, in other words, an existential crisis (Straube *et al*., 2015).

Again we ask ourselves, what loss are we talking about? It no longer matters, as it will be up to the team to recognize pain based on the assumption that patients can learn strategies to modify thoughts and beliefs, manage emotional states, and productively modify their behavior. In this way, we clarify that Artificial Intelligence contributes to the resolution of objective issues, which does not invalidate the subjectivity that constitutes being, that is, feelings and emotions will continue as individual experiences of each person.
